# RNA-Seq Analysis of Differential Splice Junction Usage and Intron Retentions by DEXSeq

**DOI:** 10.1371/journal.pone.0136653

**Published:** 2015-09-01

**Authors:** Yafang Li, Xiayu Rao, William W. Mattox, Christopher I. Amos, Bin Liu

**Affiliations:** 1 Department of Biomedical Data Science, Geisel School of Medicine, Dartmouth College, Hanover, New Hampshire, 03755, United States of America; 2 Center for Genetics and Genomics, Department of Genetics, The University of Texas MD Anderson Cancer Center, Houston, Texas, 77030, United States of America; NIH, UNITED STATES

## Abstract

Alternative splicing is an important biological process in the generation of multiple functional transcripts from the same genomic sequences. Differential analysis of splice junctions (SJs) and intron retentions (IRs) is helpful in the detection of alternative splicing events. In this study, we conducted differential analysis of SJs and IRs by use of DEXSeq, a Bioconductor package originally designed for differential exon usage analysis in RNA-seq data analysis. We set up an analysis pipeline including mapping of RNA-seq reads, the preparation of count tables of SJs and IRs as the input files, and the differential analysis in DEXSeq. We analyzed the public RNA-seq datasets generated from RNAi experiments on *Drosophila melanogaster* S2-DRSC cells to deplete RNA-binding proteins (GSE18508). The analysis confirmed previous findings on the alternative splicing of the *trol* and *Ant2* (*sesB*) genes in the CG8144 (*ps*)-depletion experiment and identified some new alternative splicing events in other RNAi experiments. We also identified IRs that were confirmed in our SJ analysis. The proposed method used in our study can output the genomic coordinates of differentially used SJs and thus enable sequence motif search. Sequence motif search and gene function annotation analysis helped us infer the underlying mechanism in alternative splicing events. To further evaluate this method, we also applied the method to public RNA-seq data from human breast cancer (GSE45419) and the plant *Arabidopsis* (SRP008262). In conclusion, our study showed that DEXSeq can be adapted to differential analysis of SJs and IRs, which will facilitate the identification of alternative splicing events and provide insights into the molecular mechanisms of transcription processes and disease development.

## Introduction

**Fig 1:**
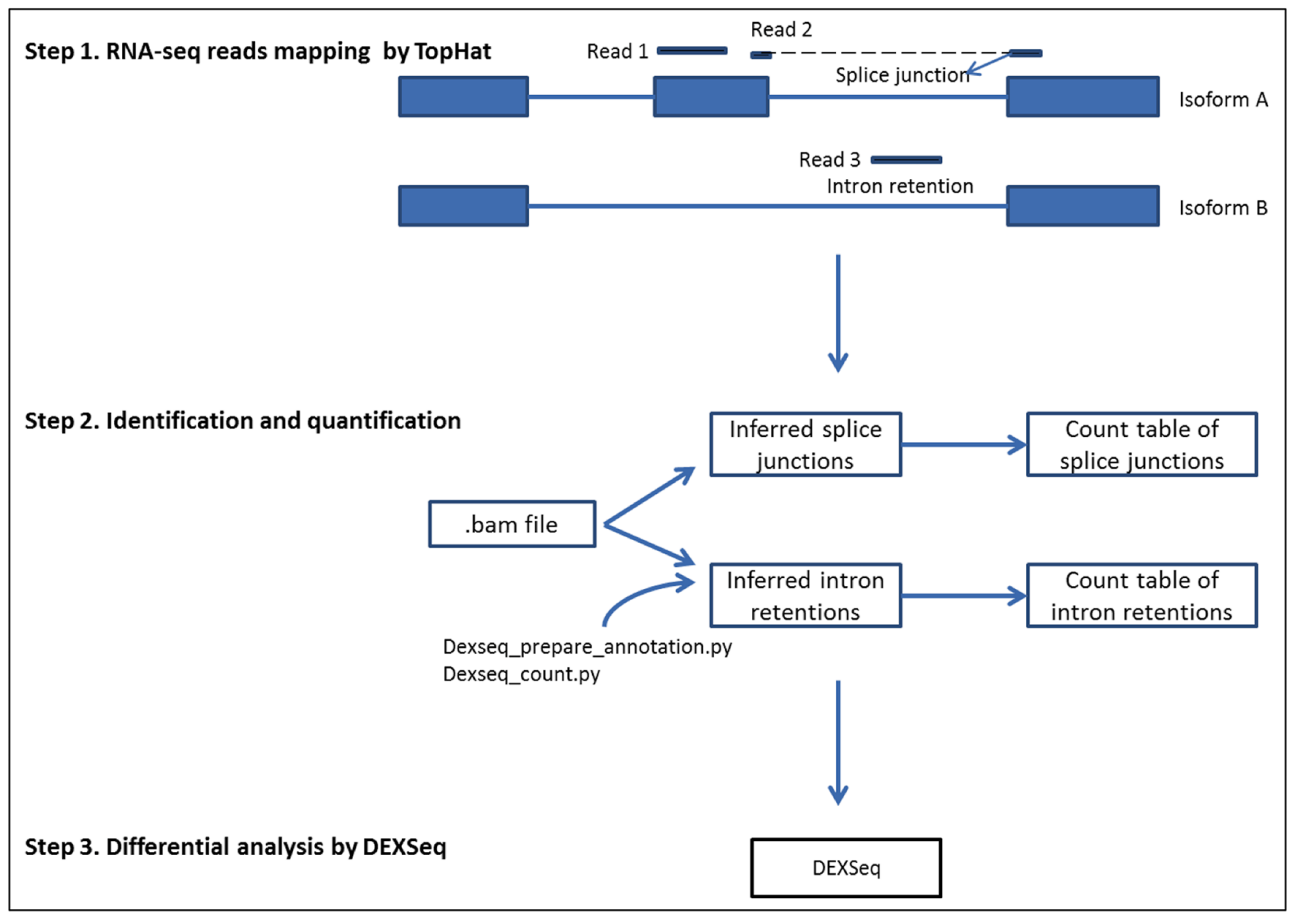
Illustration of analysis steps in differential analysis of splice junctions and intron retentions.

Alternative splicing is an essential biological mechanism that controls gene expression and increases protein diversity. Alternative splicing can be generally categorized into four major groups: (1) exon skipping, (2) alternative 5’ splicing, (3) alternative 3’ splicing, and (4) intron retention (IR) [1–3]. Intron retentions, which are believed to come from unspliced or incompletely spliced pre-mRNAs, are the rarest type of alternative splicing in mammals and account for only approximately 3% of alternate transcripts [4, 5]. Increasing evidence has shown that IRs are biologically significant and involved in disease development [6, 7]. Splice junctions (SJs) are the exon-intron connections where splicing takes place; IRs are retained intron fragments in mRNA ([1–3], Fig 1). Differential analysis of SJs and IRs is helpful in the detection of alternative splicing events.

RNA-seq technology provides a revolutionary tool for analysis of the transcriptome. It offers the opportunity to detect new genes and alternative splicing events on a genome-wide scale, which is essential for understanding development and disease mechanisms in a species [8, 9]. The vast quantities of sequencing data generated by RNA-seq experiments require robust and efficient algorithms to process and analyze the information. Different approaches have been developed for quantifying differential expression of mRNA isoforms with consideration of alternative splicing. The software tool Cufflinks/Cuffdiff can detect alternative splicing on the gene transcript level [10, 11]. Katz et al. developed the MISO model to infer estimates based on the reads that were mapped to SJs and alternative spliced exons [12]. ALEXA-seq is another method that was developed for alternative expression analysis of genetic features including exon regions, exon junctions, and intron regions [13]. Other software tools designed for alternative splicing analysis include MATS, SplicingCompass, and JUNCBASE [14–16].

DEXSeq is an R Bioconductor package that is widely used in differential expression analysis of exons [17]. DEXSeq applies generalized linear models and identifies specific exons usage with high sensitivity. We proposed using the powerful DEXSeq package for differential analysis of SJ usage and IRs after supplying it with proper read count data. In this current study, we report the applications of DEXSeq in alternative splicing analysis including IR analysis using RNA-seq data from the fruit fly *Drosophila*, the plant *Arabidopsis*, and human breast cancer.

## Materials and Methods

### RNA-seq data

RNA-seq data in *Drosophila* was downloaded from the Gene Expression Omnibus (GEO) web site with accession number GSE18508 [16, 18]. The Graveley group generated GSE18508 datasets using deep sequencing of mRNA from *Drosophila melanogaster* S2-DRSC cells that had been RNAi-depleted of mRNAs encoding RNA-binding proteins. To align with the most updated reference file, we began our analysis from the.sra files in the GSE18508 datasets. There were 203 SAM files coming from the 57 RNAi experiments and unaffected controls.

Human breast tumor RNA-seq data were downloaded from GEO with accession number GSE45419 [19]. This data set included eight replicates from benign breast lesions and estrogen receptor–positive (ER+), triple negative, and human epidermal growth factor receptor–positive (HER2+) breast tumors. We used our method to test for differential usage of SJs and introns between HER2+ samples and the other three types of samples.

RNA-seq data in *Arabidopsis* were also downloaded from GEO with accession number SRP008262 [20]. The four samples included two wild-type controls and two SKIP mutants. SKIP is a splicing factor involved in transcriptional regulation in *Arabidopsis*.

### RNA-seq analysis

We used TopHat 2.0.4, with Bowtie 2.0.0.7, and SAM tools 0.1.18.0 for RNA-seq reads mapping [21]. Reference genomes came from Ensembl BDGP5.25 for *Drosophila* data sets and Ensembl GRCh37 for the human breast cancer data set. In TopHat mapping, we used the parameter “–min-segment–intron 2” to set the minimum intron length as 2, in order to retain as many intron segments as possible. The parameter “–a/--min-anchor-length” was set as 8 to ensure that the reported SJs were supported by at least one read with at least 8 bp on each side. The other parameters were set by default in TopHat.

### Sequence motif search for splice sites

Our method provided the genomic coordinates of the significant SJs, which allowed us to conduct a sequence motif search at the flanking sequence of significant SJs by differential analysis. If the splice site was labeled as 0 bp, fragments from 5th bp to 55th bp were retrieved from both directions and submitted to the MEME suite, an online tool for motif discovery and searching, for sequence motif search [22]. The strategy of 5-bp sliding was implemented to avoid the possible conserved GT/AG site at splicing recognition sites. The frequencies of the motifs in significant SJs were compared with those in all SJs in the reference genome, and the adjusted *p*-values for multiple comparisons are reported.

### Other analytical tools used

We utilized IGV (Integrated Genomic Viewer) to visualize the genes with significant SJs or IR [23]. The functional roles and relationships of the significant genes from SJ and IR analyses were analyzed by IPA (Ingenuity Pathway Analysis). For each gene, the most significant *p*-values from the SJs were assigned as input. Although IPA lacks a database for *Drosophila*, it can provide the vertebrate homologue of each significant gene to complete the analysis; genes that did not have a vertebrate homologue were removed.

## Results

### Pipeline for differential analysis of SJ usage and IRs

Fig 1 outlines the procedure used in our study. The procedure included three steps, starting from RNA-seq mapping, preparation of count tables and references of SJs and IRs as the input file for DEXSeq, and differential analysis in DEXSeq. The sequence reads with information of SJs can be interpreted with the CIGAR string field (Column 6) in the SAM file. Knowing the start position of the mapped sequence and sequence lengths for the alignment match and skipped region, we can infer the precise start and end positions of the SJs. SJs that spanned two different genes were listed as one event in each gene. A count table for SJs was created, and SJs from the same gene were numbered sequentially as the SJ reference to mimic the exon library in DEXSeq analysis. The count table and reference file were used as input files in DEXSeq for differential analysis of SJ usage. SJ tags with a read count less than 10 were removed from differential analysis. The program was written in shell script and is available for download at http://sourceforge.net/projects/differential-sj-usage/files/.

In the IR analysis, we used the Python script dexseq_prepare_annotation.py, from the DEXSeq package, to generate the non-overlapping exon reference file, and we then converted it into a reference for non-overlapping introns. If the intron length was greater than 6 bp, 3 bp were deducted from both ends of the introns to remove the ambiguous regions. We then used dexseq_count.py to count reads at each intron fragment using the.bam file as the input file. The intron count table and intron reference file were used as the input files in DEXSeq for differential analysis of IR. Intron tags with too few counts were removed from the analysis.

### Analysis of RNA-seq data from RNAi experiments in *Drosophila*

The RNA-seq data in *Drosophila* came from the depletion of various splicing factors by RNAi experiments. With the aberrant function of splicing factors, the transcription process of genes would be affected, resulting in differential usage of SJs in the organism. In this study, significant SJs and IRs with adjusted *p*-values less than 0.05 in the differential analysis were reported. There were 57 RNAi experiments, of which 14 produced at least 15 or more significant events across the whole genome. The numbers of significant events and genes harboring those events on each chromosome are summarized in Table 1. The counts for genes and SJs indicate the multiple significant SJ events that could happen on one gene. We also noted that the number of signals was approximately proportional to the size of the chromosome. Chromosome 3R showed more alternative splicing events than did the other chromosomes. Different RNAi experiments generated very different numbers of significant SJs and IRs. CG10279 and CG8144 produced relatively large numbers of signals in both SJ and IR analyses. Information regarding the complete list of significant SJs and IRs is available in the Supporting Information (S1 and S2 Tables).

**Table 1:** Number of significant signals and genes on each chromosome.

		Chromosome						
RNAi	Gene name	2L 23.0Mbp	2R 21.1Mbp	3L 24.5Mbp	3R 27.9Mbp	4 1.4Mbp	X 22.4Mbp	Total
**Splicing Junctions analysis**								
CG10279	*Rm62*	(45,59)	(58,74)	(39,49)	(59,72)	(3,4)	(40,49)	(244,307)
CG10851	*B52*	(10,11)	(4,4)	(11,14)	(17,17)	(4,4)	(8,13)	(54,63)
CG12749	*Hrb87F*	(5,5)	(2,3)	(1,1)	(2,5)	(1,1)	(4,7)	(15,22)
CG1559	*Upf1*	(5,5)	(8,8)	(9,9)	(8,11)	(0,0)	(4,4)	(34,37)
CG32423	*shep*	(198,293)	(247,378)	(187,303)	(275,423)	(14,19)	(177,246)	(1098,1662)
CG5170	*Dp1*	(6,7)	(3,4)	(2,4)	(3,3)	(0,0)	(3,5)	(17,23)
CG6946	*glo*	(1,1)	(5,7)	(2,3)	(4,8)	(1,1)	(1,1)	(14,21)
CG8144	*ps*	(60,88)	(72,107)	(64,107)	(93,127)	(5,6)	(59,102)	(353,537)
CG8241	*pea*	(9,12)	(7,7)	(8,9)	(6,6)	(0,0)	(4,4)	(34,38)
CG8636	CG8636	(120,158)	(153,212)	(105,145)	(162,237)	(6,7)	(103,135)	(649,894)
CG8749	*snRNP-U1-70K*	(4,5)	(2,2)	(7,7)	(6,6)	(0,0)	(4,5)	(23,25)
CG8781	*tsu*	(44,58)	(47,56)	(38,47)	(59,69)	(4,4)	(44,53)	(236,287)
CG8912	*Psi*	(61,81)	(74,101)	(40,64)	(72,91)	(1,1)	(57,68)	(305,406)
**Intron retention analysis**								
CG10279	*Rm62*	(30,47)	(34,58)	(28,44)	(52,83)	(1,1)	(35,57)	(180,290)
CG10851	*B52*	(8,9)	(7,9)	(5,7)	(10,12)	(0,0)	(4,6)	(34,43)
CG11266	CG11266	(2,2)	(2,2)	(5,5)	(4,5)	(0,0)	(1,2)	(14,16)
CG12749	*Hrb87F*	(2,2)	(4,4)	(3,4)	(5,6)	(0,0)	(2,2)	(16,18)
CG32423	*shep*	(1,1)	(1,1)	(4,6)	(2,3)	(0,0)	(3,6)	(11,17)
CG6946	*glo*	(2,3)	(4,6)	(5,7)	(6,8)	(0,0)	(5,7)	(22,31)
CG8144	*ps*	(16,19)	(22,37)	(18,26)	(24,44)	(3,4)	(23,34)	(106,164)
CG8241	*pea*	(33,61)	(30,56)	(35,51)	(45,83)	(1,1)	(26,39)	(170,291)
CG8636	CG8636	(2,3)	(3,3)	(4,6)	(3,4)	(0,0)	(9,14)	(21,30)
CG8749	*snRNP-U1-70K*	(10,16)	(9,11)	(5,9)	(4,6)	(0,0)	(9,12)	(37,54)
CG8781	*tsu*	(8,11)	(10,12)	(7,8)	(5,7)	(0,0)	(5,9)	(35,47)
CG8912	*Psi*	(9,12)	(9,13)	(7,10)	(12,18)	(0,0)	(8,13)	(45,66)

For each chromosome, the left column shows the number of significant genes, and the right column shows the number of significant splicing junctions or intron retentions.

**Fig 2:**
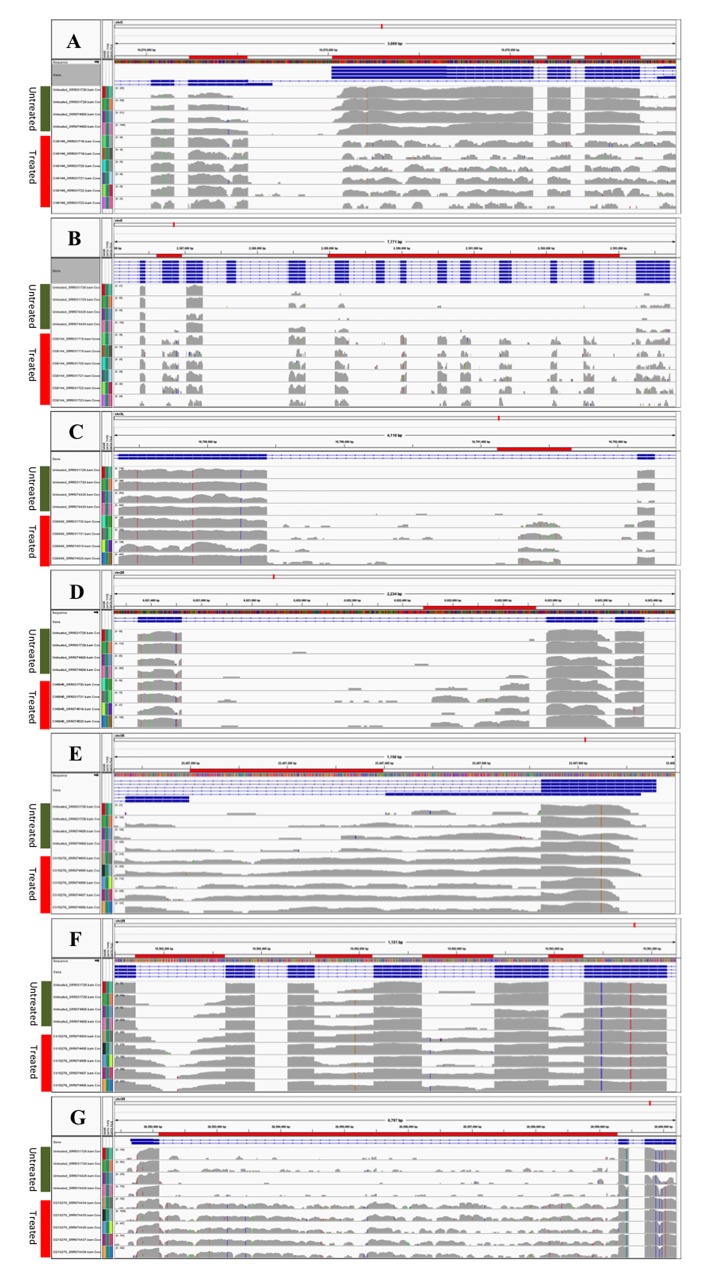
Visualization of splicing junctions and retained introns. In each panel, the first four lines denote samples from the untreated condition; the other lines denote samples from the RNAi-treated condition. The exons with differentially utilized splicing junctions are marked with red horizontal bars and the splicing junction positions are matched to the ones listed in Table 2. (A) Gene *Ant2/sesB* from CG8144 RNAi experiment. (B) Gene *trol* from CG8144 RNAi experiment. (C) Gene CG9674 from CG6946 RNAi experiment. (D) Gene *gem* from CG6946 RNAi experiment. (E), (F) and (G) Retained introns detected at *wdb*, *pde8*, and *zfh1*genes from the CG10279 RNAi experiment. The retained introns are highlighted with red horizontal bars as well.

Table 2 presents the output from the differential analysis of CG8144 and CG6946 RNAi with the genomic coordinates of SJs, adjusted *p*-value, and log2 fold change. Six significant SJs were identified in the Ant2/sesB gene in the CG8144 dataset. The first three SJs, 10,673,730–10,674,026; 10,674,155–10,674,230; and 10,674,561–10,680,893, had a higher expression in RNAi-treated samples, and the log2 fold (untreated/treated) varied from -3.8 to -5.7. The other three significant SJs, 10,676,130–10,676,206; 10,676,335–10,676,413; and 10,676,716–10,680,839, had lower expressions in the treated samples, resulting in a positive log2 fold value of around 2–3 at these three SJs. The IGV plots in Fig 2A clearly demonstrate the alternative splicing events at this region. The *Ant2* and *sesB* genes overlapped with each other and shared part of their transcripts. The untreated samples had higher expression in the first three exons of gene *sesB*, resulting in higher usage of the SJs between those exons; the treated samples had higher expression at the three exons of gene Ant2, creating more SJs at this region. The differences between untreated and treated samples were so significant that the DEXSeq produced an adjusted *p*-value of 0 at these locations. These results suggest that *ps* knockdown caused opposite effects on the *Ant2* and *sesB* genes. The CG8144 (*ps*) gene in *Drosophila* is the homologue to the RNA-binding protein genes nova-1 and nova-2 in humans [24]. A previous report found alternative splicing events at exactly the same location in the gene *ps*, identified by the use of JuncBase [16]. Our study replicated their results and confirmed the relationship of *ps* splicing factor and the *Ant2*/*sesB* gene target.

**Table 2:** Alternative splicing detected in the CG8144 and CG6946 RNAi experiment in *Drosophila*.

Position	Dispersion	*p*-adjusted	Mean	log2 fold (untreated/treated)	Gene name
**CG8144 RNAi**					
**Splice Junctions**					
10673730–10674026	0.1	0	12.8	-3.81	*Ant2; sesB*
10674155–10674230	0.08	0	17.94	-4.01	*Ant2; sesB*
10674561–10680839	0.11	0	11.96	-5.7	*Ant2; sesB*
10676130–10676206	0.06	0	53.05	2.23	*Ant2; sesB*
10676335–10676413	0.05	0	46.07	2.07	*Ant2; sesB*
10676715–10680839	0.09	3.15E-07	14.71	2.94	*Ant2; sesB*
2386455–2386688	0.24	4.96E-03	4.46	-3.22	*trol*
2386455–2387021	0.32	1.66E-03	3.31	3.62	*trol*
2386920–2387021	0.25	8.87E-05	4.28	-4.56	*trol*
2388672–2389074	0.15	5.68E-08	7.55	-4.53	*trol*
2390068–2390494	0.17	2.50E-10	6.8	-6.8	*trol*
2390628–2390809	0.3	8.15E-05	3.51	-5.85	*trol*
2390960–2391351	0.86	1.10E-03	4.5	-30.84	*trol*
2391523–2392055	0.51	2.58E-03	2.03	-29.9	*trol*
2392150–2392516	0.21	1.34E-08	6.88	-6.65	*trol*
2392666–2393244	0.44	4.62E-04	2.36	-30.21	*trol*
2387250–2395660	0.33	2.47E-06	3.23	32.06	*trol*
2392666–2395660	0.51	1.75E-03	2.02	-30.02	*trol*
**CG6946 RNAi**					
**Retained Introns**					
16789440–16792142	0.07	2.00E-08	13.38	-3.06	CG9674
6021525–6022776	0.14	2.79E-02	7	-1.67	*gem*
**Splice Junctions**					
16789439–16791272	0.44	2.60E-02	2.7	-28.77	CG9674
16789439–16791532	0.37	5.12E-03	3.26	-29.03	CG9674
6022906–6022973	0.27	1.02E-05	5.04	-27.87	*gem*
**CG10279 RNAi**					
**Retained Introns**					
23407000–23407400	0.12	4.77E-02	117.76	-1.42	*wdb*
19562145–19562324	0.26	1.74E-02	14.79	-2.41	*Pde8*
19562731–19562875	0.29	7.94E-03	5.8	-3.73	*Pde8*
19562991–19563059	0.2	1.44E-02	8.63	-2.57	*Pde8*
26592105–26599292	0.01	0.00E+00	223.79	-1.76	*zfh1*
26600422–26608977	0.02	0.00E+00	496.22	1.63	*zfh1*
**Splice Junctions**					
23394313–23405879	0.12	2.13E-02	28.59	-1.9	*wdb*
19562176–19562327	0.37	4.78E-03	3.84	-28.03	*Pde8*
26592104–26599295	0.09	4.80E-02	166.59	0.81	*zfh1*

Another gene we identified in SJ analysis was the *trol* gene in the CG8144 RNAi experiment. The untreated samples had a long SJ annotated across the ChX: 2,387,600–2,396,000 that skipped 15 exons. However, nine of the 15 exons were utilized for the transcripts in the treated samples, which created many more short SJs at this region (Fig 2B). The nine SJs in the treated samples resulted in negative values for the log2 fold change in the DEXSeq analysis; the abundant large SJs in the untreated samples explained the positive values for the log2 fold change (Table 2). The significant SJs detected in the *trol* gene provide a good illustration of exon skipping in alternative splicing events.

In additional SJ events, we detected some partially retained introns using the analytical pipeline. The CG6946 (*glo*) gene plays an important role in patterning the *Drosophila* anterior-posterior axis by functioning as both a splicing regulator and a translational repressor [25, 26]. The depleted function of CG6946 affects the transcription of many genes in the organism. Table 2 lists the two significant expressed regions within the introns of genes CG9674 and *gem* for the dataset CG6946. The two revealed IR fragments were also confirmed by integration of our SJ analysis, as retained introns would introduce new SJs. Fig 2C and 2D show that the two novel SJs were at the boundary sides of the putative IR in the CG9674 gene. In the RNAi-untreated samples, no reads were detected across the new SJs; in the treated samples, total counts of 104 and 117 per 50 million reads were detected at these two novel splice sites, respectively (S1 Fig). Another example for IR analysis is the retained region in the gem gene. The IR analysis detected an IR at Ch2L: 6021525–6022776, which was consistent with the evidence from the SJ analysis, i.e., a new SJ at Ch2L: 6022906–6022973 on the right side of the retained intron (Fig 2D). The gene gem encodes a transcription factor in *Drosophila*, and a study of the homologue of gem in honeybees showed that alternative splicing in this gene plays an important role in the regulation of worker sterility [27]. The human homologue of gem, the tfcp2 gene, acts as an oncogene for hepatocellular carcinoma [28]. Our data suggested that the gene CG6946 knockdown had an adverse effect on the transcription of CG9674 and gem genes, which may result in an aberrant development process in *Drosophila*. Fig 2C and 2D show the partially retained introns identified in the analysis, which could be novel or unannotated exons within a gene. We also identified the complete IRs for genes *wdb* and *pde8* from the CG10279 RNAi dataset (Fig 2E and 2F). There was one IR for gene *wdb* with an adjusted *p*-value of <0.05 and three IRs for gene *pde* with adjusted *p*-values ranging from 0.008 to 0.02 (Table 2).

### Sequence motif search and gene function annotation

**Fig 3:**
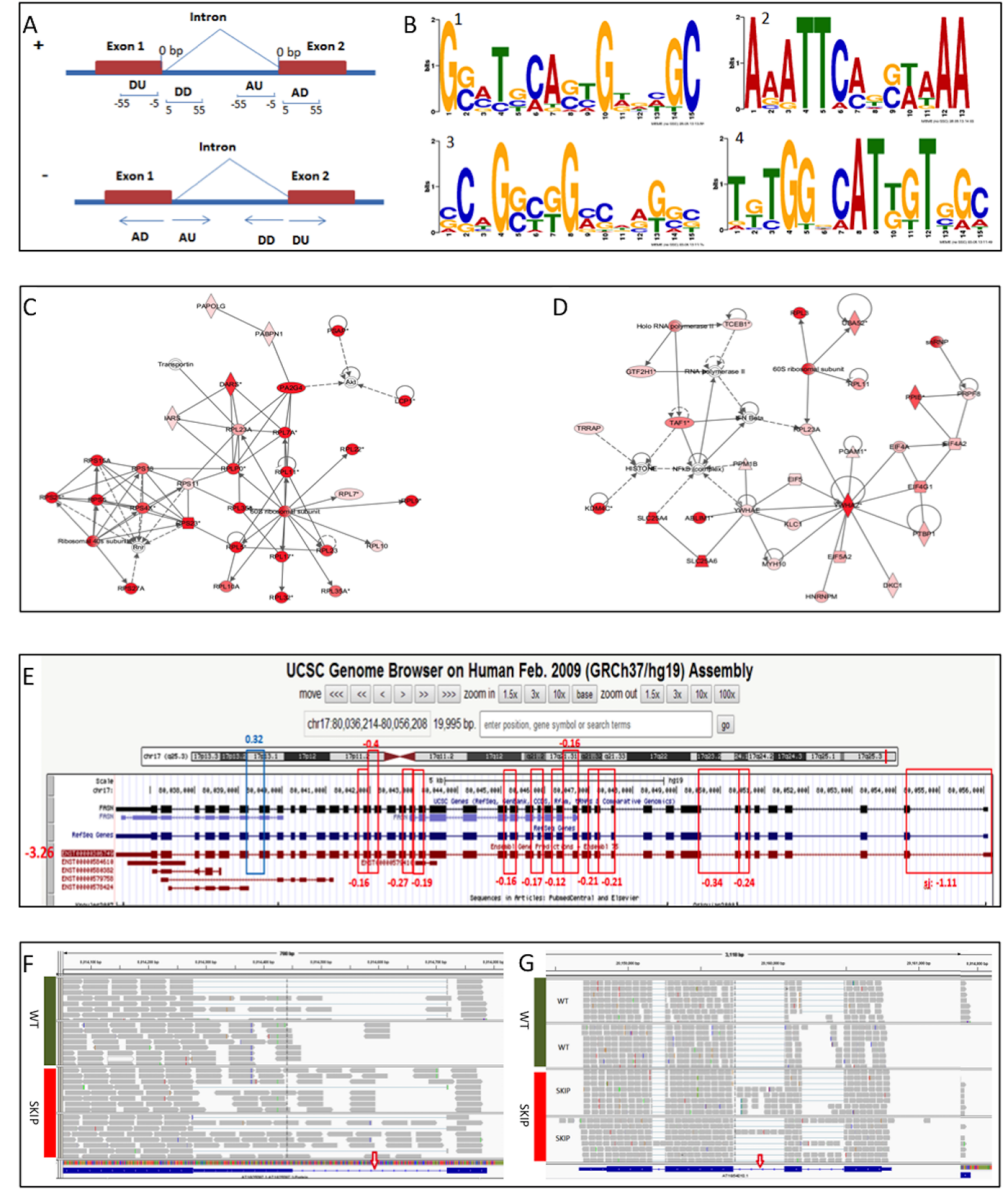
Motif analysis and multi-species comparison of splicing junctions and intron retentions. (A) Illustration of the donor upstream (DU), donor downstream (DD), acceptor upstream (AU), and acceptor downstream (AD) sequences used in the motif search. (B) Sequence motifs detected at flanking sequences of significant splicing junctions. Motif 1–4 corresponds to that in Table 3. (C) Networks from genes with significant splice junctions (SJs) in CG8144 RNAi, IPA score 59. (D) Networks from genes with significant SJs in CG10279 RNAi, IPA score 54. The color denotes the significance of the SJ within the gene: the darker the red is, the more significant the SJ in the gene is. (E) The consistent trend of the expression level of an isoform and the differential usage of its unique SJs between HER2+ and benign tumor samples. (F) and (G) Intron retentions detected in AT1G25097 and AT1G54010 genes from *Arabidopsis* RNA-seq analysis. Red arrows indicate the intron retentions.

To further depict the pattern of the splicing events, we performed the motif search with the flanking sequences of the significant SJs. Sequence motifs near splicing sites often suggest the binding recognition sites of splicing factors [29]. Finding the sequence motifs would provide researchers valuable information in understanding the alternative splicing regulation mechanism. The 50-bp flanking sequences from the significant SJs were then grouped into donor downstream, donor upstream, acceptor downstream, and acceptor upstream fragments (Fig 3A). Each group of sequences then underwent sequence motif search, and Fig 3B and Table 3 show the significant motifs detected in the CG10279, CG32423, and CG8636 RNAi datasets. The identified motifs were relatively G/C rich. A top conserved 14-bp motif, G(CG)(ACG)(TC)(GCT)(CA)(ATG)(AGC)(ATC)G(ATG)(GCAT)(ACT)(GT)C, was detected at the donor downstream of the significant SJs from the CG10279 RNAi dataset. About 6% of the significant SJs in the CG10279 knockdown contained this motif, compared with only 0.1% in the control genome. The *p*-value with Bonferroni correction was very significant at 1.14e-13.

**Table 3:** Significant sequence motifs detected at flanking regions of alternative splicing sites.

		Motif sequence	RNAi experiment		Control genome		
			N1	N2	N3	N4	*p*-adjust
1	CG10279_DD	G[CG][ACG][TC][GCT][CA][ATG][AGC][ATC]G[ATG][GCAT][ACT][GT]C	277	14	69895	130	1.14E-13
2	CG10279_DU	A[AGC][AG]TT[CA][AC][GCTA][CG][AT][ATG]AA	240	10	69895	128	7.01E-09
3	CG32423_DD	[CGA][CG][ATGC]G[GC][CTA][GT]G[ACG][CGTA][ATGC][AGC][GT][GCA][CGT]	686	28	69895	573	1.84E-09
4	CG8636_DU	[TAG][GCT][CT]G[GT][GCAT][CA]AT[TG][GT]T[TGC][AG][CAT]	691	12	69895	107	2.94E-07

N1, number of significant splice junctions; N2, number of splice junctions with motif (x); N3, number of splice junctions in whole genome; N4, number of splice junctions with motif in the whole genome.

To classify the biological functions of the genes affected by the significant SJs, we conducted pathway analysis for the vertebrate homologues by using IPA, which generated network information based on functional relationships among the genes of interest. Fig 3C and 3D illustrate the significant gene networks from the CG8144 and CG10279 RNAi datasets; the darkness of the red color indicates the level of significance. These two networks were both related to RNA post-transcriptional modification, which suggests that the inhibited expression of CG8144 and CG10279 has an adverse effect on transcription regulation. Multiple genes that have a direct or indirect relation with the depleted splicing factors were affected in the organism.

### Alternative splicing analysis in breast cancer and intron retention analysis in the plant Arabidopsis

To evaluate the application of our method to studies of human diseases, we also performed the analysis on a public human breast cancer dataset (GSE45419). Kalari et al. [19] used the Bioconductor R package CASPER to identify known splice variants by analysis of the relative abundance of isoforms in the four sample groups: HER2+, ER+, triple negative, and benign. The relative abundance of an isoform that is alternatively spliced was calculated as counts for that specific isoform divided by counts for all isoforms of the corresponding gene [19]. In our study, we confirmed 40 alternatively spliced genes by combining the three pairwise comparisons between HER2+ and the other tumor types (adjusted *p*-values <0.05) in the analysis. Fig 3E displays the significant SJs we identified in the fatty acid synthase (*FASN*) gene for the HER2+ vs. benign comparison. Although the fold change was relative small, the low adjusted *p*-values were significant and indicated the small dispersion with the sample groups. One of the FASN isoforms, ENST00000306749, indicated that SJs were reduced in HER2+ samples. The results displayed the same changing direction as the gene expression analysis by Cuffdiff that had the log2 fold change (-3.26) for HER2+ vs. benign (indicated by the red color in Fig 3E). In our study, the highlighted SJs were exclusive to the isoform ENST00000306749. Consistently, DEXSeq reported decreased usage of these SJs in the HER2 samples. Varied expression of FASN has been reported in HER2+ breast cancer [30, 31]. Our findings should aid in increasing our understanding of the differential expression of a specific isoform detected during breast cancer development. The motif analysis also demonstrated that our method could help investigate the molecular mechanism of the differential expression, which could be due to changes in splicing factors or specific splicing motifs.

Although rare in mammals, IR is very common in plants [32–34]. We therefore performed IR analysis on the plant *Arabidopsis* using public RNA-seq data from the GEO SRP008262 datasets, with wild-type controls and SKIP mutants. Using a cutoff of 0.2 for the adjusted *p*-values, we identified 157 retained intron regions that were not expressed in any annotated transcripts (S3 Table). Fig 3F and 3G display the RNA reads at the IRs identified in AT1G25097 and AT1G54010; *p*-values were 1.6E-03 (adjusted *p*-value 0.17) and 7.0E-04 (adjusted *p*-value 0.11), respectively.

## Discussion

Alternative splicing plays an important role in cell differentiation and development, and it is also involved in cancer development and progression [35, 36]. Quantitative evaluation of alternative splicing is essential for understanding disease mechanisms. The computational approach based on SJs and IRs should be robust enough to detect alternative splicing events. In this study, we applied the DEXSeq tool to our SJ and IR analyses. Unlike alternative splicing analysis using Cufflinks/Cuffdiff, our method can output the genomic coordinates that pinpoint the location of the junctions. This is especially useful when researchers want to perform downstream analyses such as sequence motif searches. The depletion of the transcription factors in *Drosophila* has a tremendous effect on the gene transcription process. Our method detected significant SJs for genes such as *Ant2*/*sesB* and *trol*. These findings are substantiated by previous reports of alternative splicing [24], suggesting that our method can be a powerful tool in SJ analysis.

Intron retention is the rarest type of alternative splicing in mammalian genomes and was recently found to be related to disease development [6, 7]. IR is believed to occur when introns are not removed from the RNA transcripts, causing the fragments to be retained as part of an exon in the mRNA. Our analytical method provides a convenient tool to detect IR events in alternative splicing. The IRs detected in this study included both partial IRs, which behave like novel or unannotated exons, and canonical IRs, which consist of whole introns retained between two adjacent exons. For the retained intron region in the gene gem, the novel splice site was detected only on the right side of the IR (Fig 2D). It is possible that the other side of the IR is GC-rich and difficult to sequence. A higher read depth of RNA-seq will improve the analytical power of our method, especially for the sequencing of “cold spots.” The IR analysis was based on an annotated intron reference file and could detect the partially retained intron within the annotated intron. In this case, the exact boundaries of the partially retained introns cannot be defined. SJ analysis can supplement the boundary information regarding the retained fragment, especially when the partial retention produces novel SJs. Thus the combination of differential SJ usage and IR analysis will provide a powerful tool for identification of IRs in the genome.

One plausible explanation for IRs with low read counts is that the weak SJs flanking the IRs may not be properly recognized due to the changes in gene expression from RNAi depletion of an RNA transcription factor [37, 38]. Identification of the potential sequence motifs associated with IRs would provide us information about this aberrant process. The conserved sequence motifs detected at the flanking sequences of SJs in this study suggest that some consensus sequences do exist and that they may indicate some potential intronic and exonic *cis*-elements (Fig 3B). These consensus sequences may play an important role as the interactive targets for splicing factors that have been knocked down by RNAi.

In this study, we were interested in identification of differential SJ usage within the known genes. We first mapped the RNA-seq reads to the reference genome by TopHat and then created an SJ library based on RNA-seq mapping results. Researchers interested in SJ usage in novel genes may start with mapping the sequence reads without supplying an annotation file. To generalize the use of our method, we applied it to RNA-seq data from human breast cancer and the plant *Arabidopsis*. Our results were comparable with those found using Cufflinks/Cuffdiff, a popular program for differential gene expression and alternative splicing analysis. Researchers are usually interested in the differential gene expression associated with a particular disease condition, and our method provides support for differential expression from a different perspective, i.e., alternative SJ usage. Our study demonstrated that alternative splicing is an important regulatory mechanism in the transcription process and in human diseases. Our method is efficient in alternative splicing analysis owing to the identification of differential SJ usage and IRs and should aid in furthering our understanding of the complex transcription process and disease development mechanisms.

## Supporting Information

S1 Fig Visualization of more splicing junctions and intron retentions with IGV. (DOCX)

S1 Table The list of significant splicing junctions. (XLS)

S2 Table The list of significant intron retentions including partially retained introns. (XLS)

S3 Table The result from the intron retention analysis for the plant *Arabidopsis*. (XLSX)
