# Supplementary material for: RNA-Seq Analysis of Differential Splice Junction Usage and Intron Retentions by DEXSeq
Source: PLoS One. 2015 Sep 1;10(9):e0136653. doi: 10.1371/journal.pone.0136653 (PMC4556662; doi:10.1371/journal.pone.0136653)
Supplement: S1 Fig — (DOCX) [file pone.0136653.s001.docx]

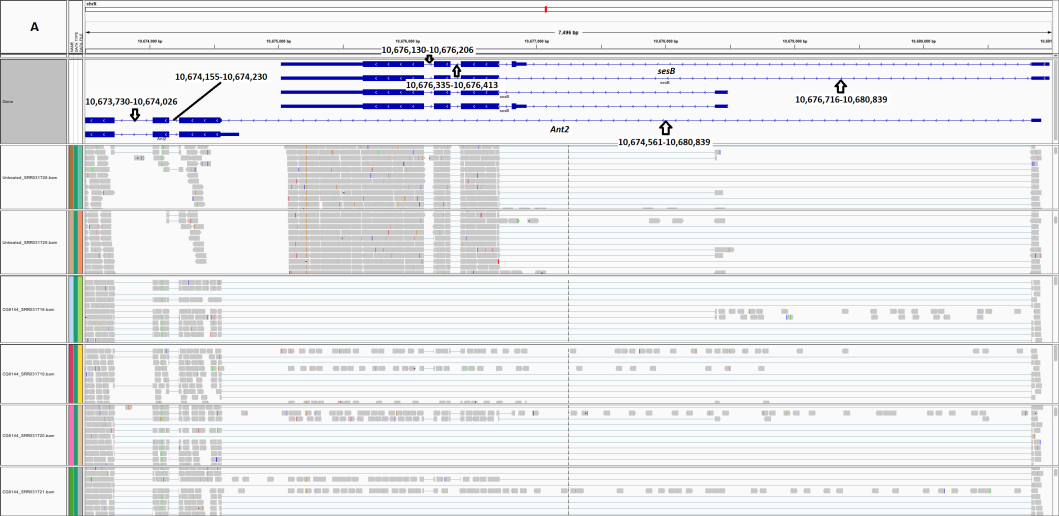


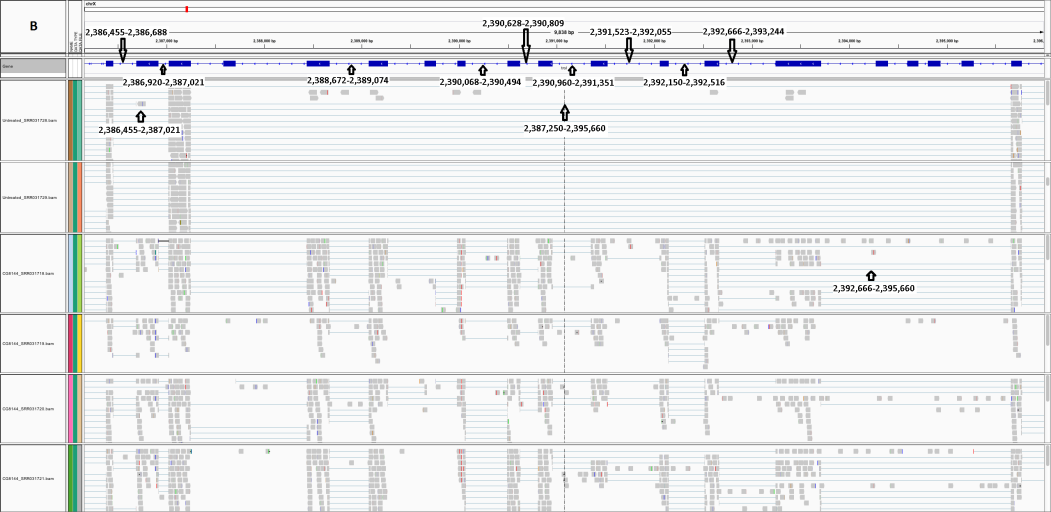


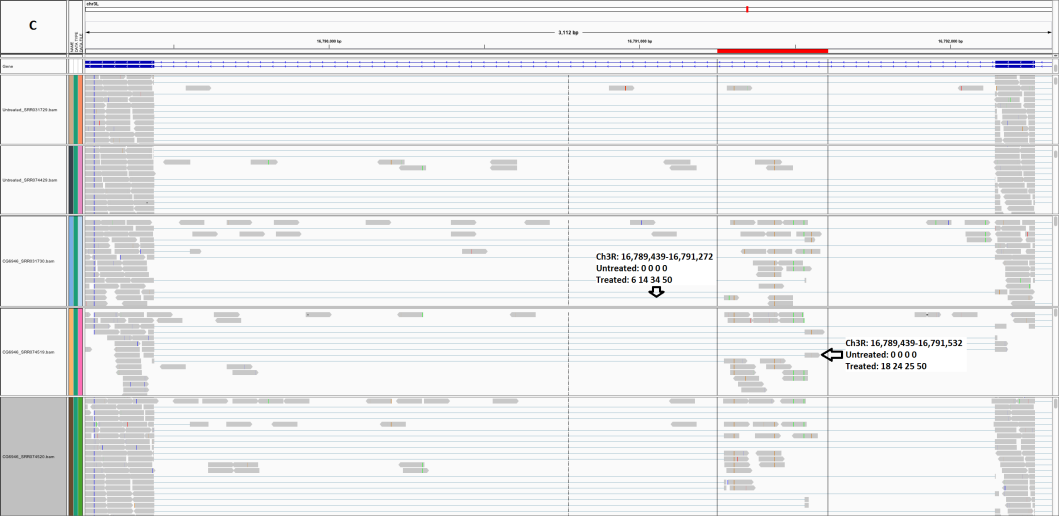


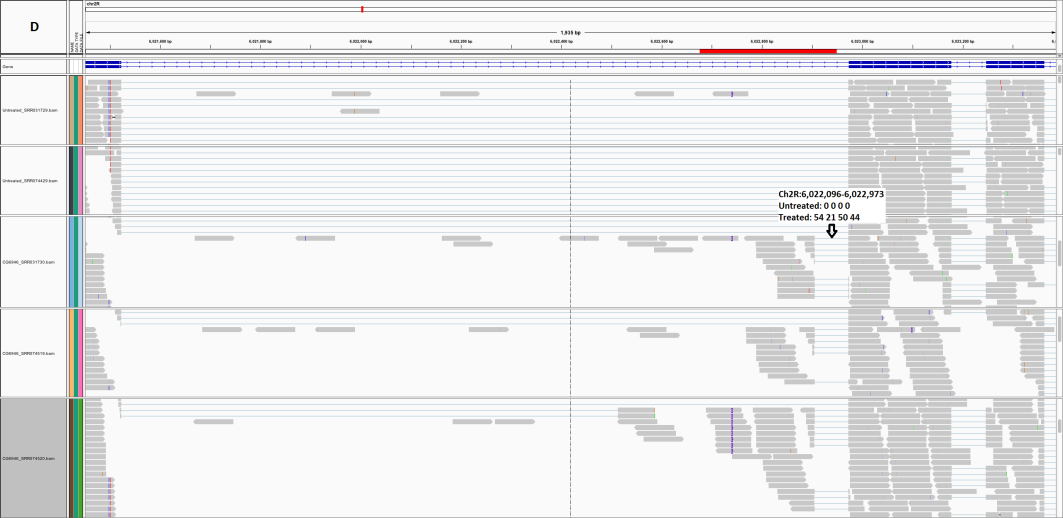


Figure S1. Visualization of more splicing junctions and intron retentions with IGV

Two untreated and three treated samples were displayed in IGV plots due to the limited window size. In each plot, line 1-2 denote samples from untreated condition; line 3-5 denote samples from RNAi treated condition. Plot A-B, CG8144 RNAi experiment. Plot A, *Ant2/sesB*; plot B, *trol* gene. The splicing junctions labeled with positions are matched to the ones listed in Table 2. Plot C-D, CG6946 RNAi experiment. Plot C, CG9674 gene; plot D, *gem* gene. The retained introns are highlighted in red. The number of splicing junctions per 50,000,000 reads at the retained intron is shown for the samples in the figures. The splicing junctions labeled with positions are matched to the ones listed in Table 2.
